# ADHD as a disorder of operational capacity: a buffered-state framework for sustained engagement instability

**DOI:** 10.3389/fpsyt.2026.1786541

**Published:** 2026-07-16

**Authors:** Tianlai Tang, Christine Dong

**Affiliations:** Nashville Brain Institute, Nashville, TN, United States

**Keywords:** ADHD, affective dysregulation, engagement instability, executive function, metastability, operational capacity, sustained attention

## Abstract

Attention-deficit/hyperactivity disorder (ADHD) is commonly conceptualized as a disorder of executive dysfunction or impaired motivation. In clinical practice, however, many individuals with ADHD describe a different core difficulty: they can initiate tasks appropriately, understand goals, and exert effort, yet struggle to remain engaged as time-on-task and internal strain accumulate. These failures are often abrupt, ego-dystonic, and strongly context-dependent, posing challenges for models based solely on static deficits or gradual depletion. Building on this phenomenology, we propose a constraint-level framework that conceptualizes ADHD as a disorder of operational capacity—the finite tolerance for sustaining stable mental engagement under cumulative cognitive and emotional demand. Within this framework, sustained engagement is modeled as a buffered state that remains stable while cumulative load is within tolerance but becomes increasingly fragile near saturation and may undergo nonlinear collapse into disengagement. We formalize this governing constraint as Operational Economy (OE)—a regulatory, not energetic, constraint principle—and describe a pre-volitional Global Switching Framework (GSF) that mediates rapid state transitions once engagement stability is exceeded. This formulation integrates and extends cognitive-energetic and state-regulation accounts while explicitly differentiating the OE/GSF framework from these predecessors by its emphasis on threshold-dependent dynamics, bounded tolerance, and asymmetric re-engagement. This framework offers a coherent account of clinical features such as intact initial performance with late-stage disengagement, pronounced time-on-task effects, and context-dependent hyperfocus. The framework generates falsifiable predictions regarding sensitivity to cumulative demand, stress-related acceleration of disengagement, pharmacological effects that preferentially extend engagement endurance rather than normalize peak executive performance, and individual differences across ADHD subtypes and comorbid presentations. These predictions are consistent with existing empirical findings on time-dependent variability in ADHD and provide a basis for future experimental validation.

## 1 Introduction: the clinical problem of sustained engagement

### 1.1 The ADHD paradox in everyday psychiatric practice

Attention-deficit/hyperactivity disorder (ADHD) is among the most frequently encountered neurodevelopmental conditions in psychiatric practice, yet it remains conceptually difficult to explain in a manner that fully aligns with everyday clinical observation (1). Individuals with ADHD often demonstrate intact intelligence, preserved insight, and a clear understanding of task requirements. They are typically able to initiate tasks appropriately and may perform comparably to peers in brief, structured, or highly supervised settings.

Despite this, they frequently struggle to remain mentally engaged over time, particularly when tasks are prolonged, cognitively demanding, monotonous, emotionally loaded, or loosely structured (2). Clinicians commonly hear a strikingly consistent description from patients: “I know what I’m supposed to do, I start doing it, but I just can’t stay with it” (3). This clinical pattern is consistent with experimental findings demonstrating increased reaction time variability and the emergence of attentional lapses during sustained tasks in ADHD, particularly under extended time-on-task conditions (2).

This failure is rarely experienced as intentional or motivational. Instead, disengagement is typically described as abrupt, involuntary, and ego-dystonic, with patients reporting that their focus suddenly “slips” or fragments despite sustained effort (3). Importantly, these failures are often nonlinear rather than gradual, occurring after a period of intact performance and producing patterns that are difficult to reconcile with linear accounts of fatigue, effort depletion, or willpower failure.

Further clinical regularity deepens this paradox. Engagement in ADHD is often restored transiently under conditions of urgency, novelty, emotional salience, or external pressure, even when it cannot be sustained under ordinary goal-directed conditions. This pattern—strong responsiveness to immediate pressure alongside unreliable maintenance of goal-driven activity—suggests not a global deficit of executive ability, but a temporal and regulatory instability in how engagement is deployed over time.

Taken together, these observations raise a central question: how can individuals who understand their goals, value task completion, and at times demonstrate intense focus nonetheless fail to sustain engagement reliably in everyday life? Why do symptoms fluctuate so strongly with time-on-task, emotional load, stress, and task structure?

### 1.2 Limits of executive-function and motivation-based explanations

Dominant theoretical models of ADHD emphasize deficits in executive functions such as inhibition, working memory, planning, and cognitive flexibility (4–6). These models have been influential and empirically productive, particularly in explaining performance on brief cognitive tasks. However, they are less well suited to explaining why individuals with ADHD may perform adequately at task onset yet fail to sustain engagement as cognitive and emotional demands accumulate over time.

Executive-function frameworks primarily characterize control as a momentary capacity rather than as a state whose stability must be maintained across time and demand. As a result, phenomena such as sudden disengagement, pronounced time-on-task effects, and asymmetry between disengagement and re-engagement are often treated as secondary or epiphenomenal rather than as central features of the disorder (7–9).

Motivation- and reward-based accounts attempt to address time-dependent variability by emphasizing altered sensitivity to incentives, delay, or boredom. While these models help explain context-dependent engagement and responsiveness to external structure, they do not fully account for disengagement that occurs despite strong motivation, meaningful goals, or explicit incentives, nor for the involuntary and pre-volitional quality of engagement collapse commonly reported by patients (3, 10, 11).

A closer conceptual precursor is provided by cognitive-energetic and state-regulation models, which emphasize the modulation of arousal, activation, and effort allocation under varying task demands and contextual conditions (12–14). These accounts have been instrumental in characterizing time-on-task effects and context sensitivity in ADHD. However, they primarily conceptualize regulation as continuous adjustment within a dynamic range, in which performance varies as a function of fluctuating energetic states.

A related line of work in dynamical-systems neuroscience conceptualizes brain function in terms of metastability, attractor dynamics, and large-scale state transitions (e.g., 15, 16). These frameworks provide important insights into how neural systems transition between coordinated states and how instability may emerge in psychiatric conditions. However, they are typically formulated at the level of neural population dynamics and do not explicitly specify the boundary conditions under which sustained task engagement fails under cumulative cognitive and emotional demand.

Although the present framework shares conceptual territory with each of these models, it differs from them in several mechanistically important respects. Cognitive-energetic models (14) and state-regulation accounts (13, 17) conceptualize performance variation as continuous modulation of arousal, effort, and activation within a dynamic range; the Operational Economy/Global Switching Framework (OE/GSF) diverges by proposing that engagement stability is bounded by tolerance limits that, when exceeded, produce threshold-dependent, nonlinear collapse rather than smooth degradation. Critically, these tolerance limits are not equivalent to energetic resources: neuroenergetic accounts (18) locate ADHD in dopaminergic insufficiency and metabolic underactivation, whereas OE is explicitly a regulatory constraint principle—it governs when stable engagement transitions to instability, not how much metabolic fuel is available. Metastability and attractor-based models (15, 16) describe neural population dynamics in terms of attractor landscapes and large-scale transitions; OE/GSF translates analogous dynamical principles into clinically-specified boundary conditions for engagement failure under demand, bridging neural and behavioral levels without presupposing a specific circuit implementation. Together, these distinctions generate unique predictions that are not derivable from prior frameworks without additional assumptions: (1) engagement failure is more strongly predicted by cumulative demand rate than by instantaneous task difficulty; (2) re-engagement is asymmetrically slower than disengagement, reflecting the need to restore buffering reserve before stable engagement can resume; and (3) pharmacological and contextual interventions extend engagement endurance by shifting tolerance limits rather than by enhancing peak executive capacity or altering motivational salience. These predictions are empirically distinguishable and testable with existing behavioral paradigms.

The present framework builds on these perspectives by introducing a constraint-level formulation in which engagement stability is bounded by finite tolerance limits. Within this account, failure of engagement reflects a threshold-dependent transition once cumulative load exceeds capacity, rather than continuous degradation within a dynamic range. This formulation translates dynamical systems principles into clinically observable patterns of engagement failure, while generating distinct predictions regarding abrupt disengagement, demand-rate sensitivity, and asymmetry between disengagement and re-engagement.

Recent extensions of state-regulation accounts using event-rate manipulations and physiological measures such as pupillometry further support the role of dynamic arousal and effort allocation in ADHD, particularly under varying temporal demands and task structures (19, 20). These findings are consistent with state-regulation accounts of ADHD but also suggest that performance instability may not be fully explained by continuous modulation of arousal or effort, indicating the need for additional mechanisms governing state stability and transition.

In addition, the framework formalizes a pre-volitional switching process—the Global Switching Framework (GSF)—that governs rapid state transitions once engagement stability is exceeded. This introduces an explicit mechanism for disengagement that is not reducible to motivational withdrawal or executive failure. The model further predicts asymmetry between disengagement and re-engagement, reflecting the need to restore buffering conditions before stable engagement can resume.

Together, these elements extend existing energetic and state-regulation models by incorporating bounded capacity, threshold-dependent state transition, and asymmetrical recovery dynamics. This framework is intended as a constraint-level formulation that complements existing process-based accounts rather than replacing them.

### 1.3 A missing constraint-level perspective on sudden mental state change

Clinical observations across psychiatric conditions suggest that mental states can remain stable for extended periods and then fail abruptly once tolerance limits are reached, rather than degrading gradually. Such threshold-like transitions are well described in physiology, stress regulation, and complex adaptive systems, yet remain under-theorized in psychiatric models of cognition and behavior.

In ADHD, this manifests as state-dependent failures of maintenance: attention, organization, emotional regulation, and behavioral inhibition may be generated successfully but cannot be reliably sustained. Disorganization, for example, often reflects not an inability to organize per se, but a failure to maintain sequencing, correction, and planning over time, resulting in progressive entropy rather than fixed incapacity.

Despite the ubiquity of these phenomena in clinical practice, psychiatry lacks a phenomenology-grounded, constraint-level framework capable of explaining how mental states remain stable under sustained demand, how stability limits are reached, and why breakdown often occurs abruptly rather than gradually across domains and contexts.

### 1.4 The present framework: sustained engagement as a buffered state

The present article addresses this gap by proposing a constraint-level framework that conceptualizes sustained mental engagement as a buffered state with finite tolerance for cumulative demand. We refer to the governing constraint as Operational Economy (OE)—a regulatory principle specifying how stability is bounded under cumulative demand, and explicitly not an energetic or metabolic construct—and to the pre-volitional state-transition process mediating disengagement as the Global Switching Framework (GSF). The distinction between regulatory and energetic accounts is important: OE does not posit that ADHD involves insufficient fuel or metabolic underactivation, but rather that the tolerance limits governing when engagement transitions to instability are reduced, independently of moment-to-moment energetic supply.

Engagement may remain stable despite increasing load up to a limit, beyond which instability emerges and disengagement may occur nonlinearly, often prior to conscious choice. Re-engagement typically requires reduction of load or restoration of buffering conditions, resulting in a characteristic asymmetry between disengagement and recovery.

Although the framework may have broader applicability, the present article focuses on ADHD as a condition in which engagement operates chronically near its stability limit, biasing regulation toward pressure- and salience-driven modes over sustained goal-directed control. The present contribution lies not in identifying time-on-task effects or context sensitivity, which are well established, but in providing a unified constraint-level formulation that explains these phenomena through finite tolerance, nonlinear collapse, and pre-volitional switching dynamics within a buffered system. The present framework aligns with emerging trends in computational psychiatry by formalizing behavior in terms of system dynamics and constraint-based stability, offering a bridge between phenomenological observation and computational modeling approaches.

**Figure 1:**
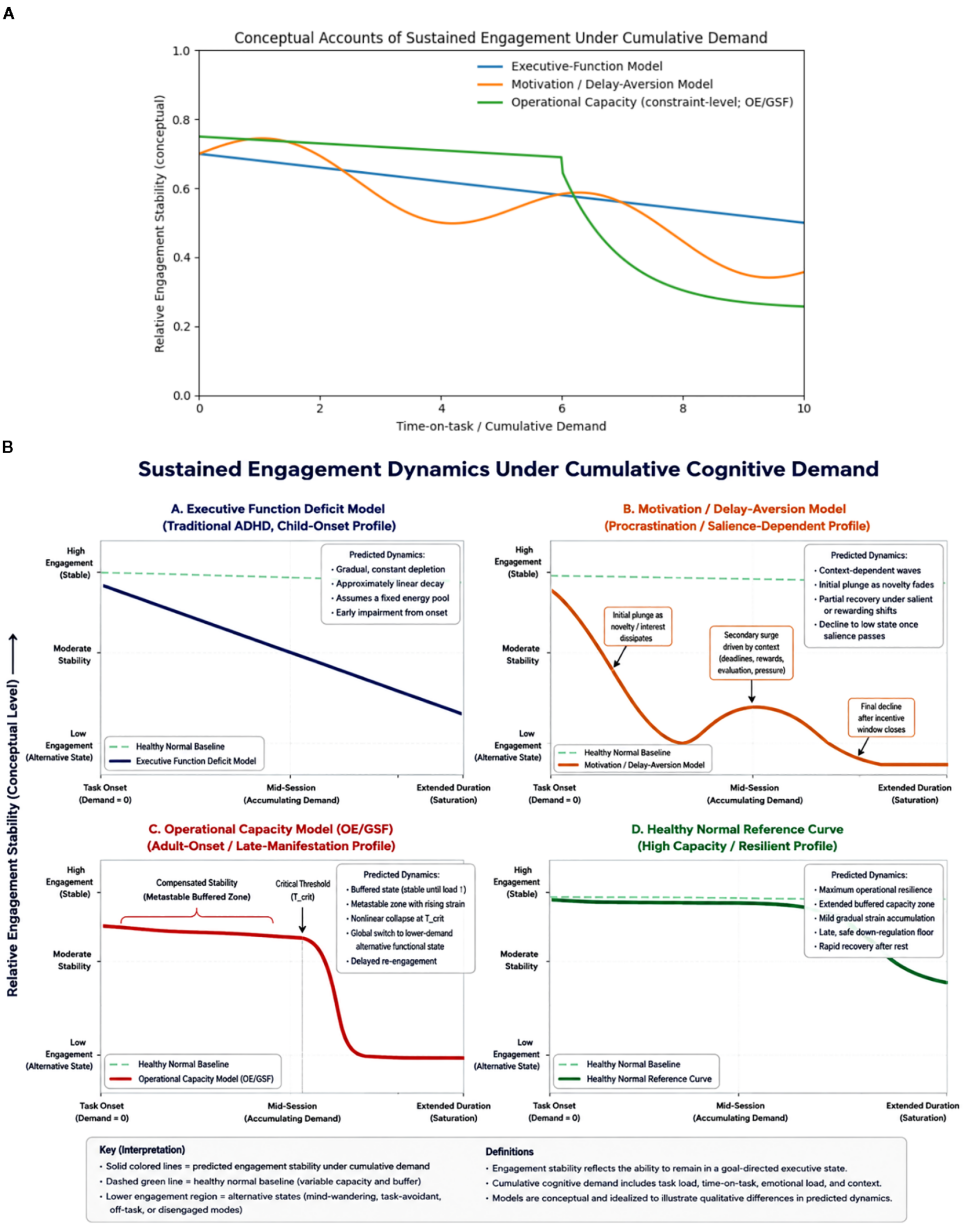
**(A)** Conceptual accounts of sustained engagement under cumulative demand. Schematic illustration comparing three major explanatory accounts of sustained mental engagement as a function of time-on-task or cumulative demand. Executive-function deficit models predict a gradual, approximately linear decline in engagement stability. Motivation- and delay-aversion models predict context-dependent fluctuations with partial recovery under salient or rewarding conditions. The operational-capacity framework (Operational Economy/Global Switching Framework; OE/GSF) conceptualizes engagement as a buffered state that remains relatively stable until cumulative demand approaches a tolerance limit, at which point engagement undergoes a nonlinear collapse followed by transition into an alternative, lower-demand state. The x-axis represents time-on-task or cumulative demand on an arbitrary scale. The y-axis represents relative engagement stability at a conceptual level rather than a specific behavioral or neural metric; in practical terms, engagement stability correlates with behavioral indices such as response time consistency, accuracy maintenance, and attentional lapse frequency. BC = Buffer Capacity (the maximum cumulative load the engagement system can absorb while maintaining stable function); BL = Buffering Load (cumulative cognitive and emotional demand integrated over time-on-task); BR = Buffering Reserve (BR = BC − BL; the remaining margin before instability is triggered). The nonlinear collapse in the OE/GSF curve corresponds empirically to the abrupt, late-stage increases in response time variability and attentional lapse frequency documented in extended sustained-attention paradigms in ADHD (2, 21). The curves are conceptual and are not intended to represent specific empirical functions, but rather to illustrate differences in predicted engagement dynamics across models. **(B)** Predicted engagement trajectories under cumulative cognitive demand across competing theoretical models of ADHD and typical development. The y-axis represents relative engagement stability (conceptual level), reflecting the ability to maintain goal-directed executive engagement, while the x-axis represents increasing cumulative cognitive demand over time. Dashed green lines indicate the healthy normative reference trajectory. Panel A illustrates the Executive-Function Deficit model, which predicts persistent impairment and gradual decline in engagement as task demands accumulate. Panel B illustrates the Motivation/Delay-Aversion model, characterized by context-dependent fluctuations, including transient recovery in response to salient incentives, deadlines, or rewards. Panel C illustrates the Operational Capacity (OE/GSF) model proposed in the present framework, in which engagement remains relatively stable within a buffered, metastable zone until cumulative load exceeds a critical threshold (T_0_crit_1_), resulting in abrupt nonlinear disengagement and transition to a lower-demand functional state. Panel D illustrates a high-capacity healthy reference profile characterized by extended buffering reserve, gradual strain accumulation, and adaptive down-regulation only under prolonged demand. The curves are conceptual illustrations intended to highlight qualitative differences in predicted engagement dynamics and are not empirical functions.

**Figures 1A, B** schematically contrast this operational-capacity account with executive-function and motivational models, highlighting differences in how sustained engagement, time-on-task effects, and abrupt disengagement are conceptualized. **Figure 1A** presents a three-curve conceptual schematic; **Figure 1B** presents the full four-panel model comparison including a healthy normative reference.

## 2 Mental regulation as a buffered system

Psychiatric theory frequently invokes constructs such as stress tolerance, cognitive load, emotional regulation, resilience, and social support, yet rarely formalizes the shared regulatory principle underlying them. When buffering remains informal, it risks appearing intuitive but theoretically diffused. When articulated explicitly, however, buffering can be understood as a lawful regulatory system—one that is layered, state-dependent, capacity-limited, and prone to instability near tolerance limits. These properties characterize mature models of regulation across physical, biological, and social domains.

In the present framework, mental buffering refers to the set of internal and external regulatory processes that preserve engagement stability under cumulative cognitive, emotional, and contextual demand by absorbing perturbation, delaying instability, and preventing premature state transition. This definition is functional rather than mechanistic, transdiagnostic in scope, and extensible across clinical contexts. Sustained mental engagement exhibits these properties and is therefore best understood as a buffered state rather than a static capacity (3).

### 2.1 Buffering as a general principle of stability

Across physical, biological, and social systems, stability under continuous input is maintained through buffering. Buffered systems absorb ongoing perturbation while preserving functional states within a finite tolerance range. This principle is evident in domains ranging from acid–base regulation to metabolic, thermal, and stress control.

Buffered systems share four defining properties: (1) dependence on cumulative load rather than instantaneous input; (2) finite tolerance rather than unlimited capacity; (3) saturation as limits are approached; and (4) nonlinear collapse once tolerance is exceeded.

Without buffering, sustained engagement would either degrade continuously with increasing demand or fail immediately. Buffering is therefore required to explain both persistence under load and abrupt breakdown—features that are central to the clinical phenomenology of ADHD.

### 2.2 Layered buffering in mental regulation

Mental regulation operates across multiple timescales and therefore relies on layered buffering mechanisms. Stability must be preserved both during moment-to-moment task engagement and across longer horizons of identity, meaning, and continuity. However, these layers do not contribute equally to psychopathology in ADHD.

At a long timescale, self-integrative buffering supports coherence of identity, values, and narrative meaning. This layer is typically preserved in ADHD and is not the primary source of functional impairment. Individuals with ADHD generally retain insight, goal valuation, and a stable sense of self, even in the presence of marked day-to-day dysfunction.

At an intermediate timescale, operational buffering stabilizes sustained engagement during ongoing activity by maintaining attention, goal persistence, inhibition, monitoring, sequencing, and emotional regulation. This layer integrates endogenous processes (e.g., attentional holding, affect tolerance) with exogenous and interpersonal scaffolding (e.g., structure, supervision, external pacing). Disruption at this level produces failures of sustained engagement despite intact understanding, planning ability, and intention. This operational layer is the primary focus of the present framework and the principal locus of impairment in ADHD.

At a background level, neuroenergetics and contextual buffering constrain engagement indirectly through metabolic support, environmental predictability, routine, and role clarity. Although these factors modulate engagement stability and treatment response, they are not treated here as primary explanatory mechanisms (18, 22).

An additional form of exogenous buffering includes pharmacological or chemical supports that may transiently expand tolerance for cumulative demand. Such supports redistribute operational load rather than repairing buffering dynamics and may involve tradeoffs in endogenous regulation over time. Across layers, buffering mechanisms differ in flexibility and cost: endogenous operational buffering is rapid, but capacity-limited, whereas exogenous buffering extends tolerance at the expense of increased dependence or downstream strain.

### 2.3 Sustained engagement as a buffered mental state

Sustained engagement is not a static ability but a dynamically regulated state. Maintaining engagement requires continuous integration of goals, suppression of competing impulses, monitoring of progress, error correction, and regulation of emotional responses, all of which impose ongoing cognitive and emotional demand (23).

Critically, these demands accumulate over time rather than resetting with each moment of effort. Buffering allows engagement to persist despite transient fluctuations, but as cumulative demand approaches tolerance limits, engagement becomes increasingly fragile. The central issue is therefore not task difficulty or effort intensity per se, but stability under cumulative load.

As buffering capacity is approached, engagement often enters a metastable regime in which performance appears intact yet becomes highly sensitive to perturbation. In this regime, small interruptions, emotional shifts, or minor increases in demand can precipitate disproportionate disengagement. This dynamic explains why engagement may appear stable until it suddenly is not, producing inconsistent performance and heightened sensitivity to stress.

### 2.4 Core components and lawful behavior of the engagement buffer

The engagement buffer comprises three interacting components.

#### 2.4.1 Accumulating operational load

Cumulative cognitive and emotional demand arising from sustained attention, integration, inhibition, monitoring, sequencing, and emotional regulation. Load increases with time-on-task and scales with task demand rate. Stress, ambiguity, and emotional tension accelerate accumulation (24).

#### 2.4.2 Buffer capacity (operational capacity)

The maximum cumulative load that can be absorbed while maintaining stable engagement. Operational capacity is not a consumable resource or a measure of effort; it is a tolerance threshold governing engagement stability across time.

#### 2.4.3 Automatic release mechanism

When cumulative load exceeds capacity, engagement becomes unstable and is released automatically. Disengagement occurs prior to conscious choice and reflects lawful system behavior rather than motivational withdrawal. This release is implemented by a pre-volitional switching process formalized in Section 3.

Buffering is not directly observable, but its operation is revealed through characteristic behavioral signatures. Stable early performance reflects intact buffering, whereas abrupt disengagement signals saturation. Hyperfixation reflects over-stabilization of engagement under high salience; procrastination reflects anticipatory avoidance of buffer exhaustion; disorganization reflects failure of ongoing maintenance and correction; substance use reflects recruitment of external buffering; and burnout reflects chronic buffer depletion.

At a formal level, buffering behavior is governed by recurrent parameters: buffer capacity, load accumulation rate, recovery rate, internal leakage (inefficiency or noise), threshold behavior, and asymmetry between disengagement and re-engagement. These parameters impose structural constraints: buffering is finite, rate-dependent, history-dependent, and asymmetric.

These constructs differ importantly from related concepts in the cognitive literature. Buffering load (BL) differs from cognitive load as traditionally conceived (e.g., 25), which refers to instantaneous processing demands on working memory; BL is explicitly cumulative and time-integrated, such that two tasks with identical moment-to-moment cognitive demands may produce different BL trajectories depending on duration and demand rate. Operational capacity (OC) differs from “effort” in that effort denotes a volitional investment of regulatory resources directed at a specific goal, whereas OC is a structural tolerance threshold that constrains when engagement stability is compromised, independent of conscious intent. Operational bandwidth (OB) differs from executive control in that executive control refers to the momentary deployment of regulatory operations such as inhibition, planning, and monitoring; OB describes the functional margin within which those operations can be sustained without triggering state collapse. Conceptually, BL accumulates in units of demand-time (the product of demand rate and duration), OC defines the upper boundary of tolerable BL in the same units, and BR quantifies the remaining margin (BR = OC − BL). While these constructs are not directly observable, behavioral proxies—including time-to-disengagement, intra-individual response time variability, attentional lapse clustering, and re-engagement latency—provide indirect indices of underlying buffering dynamics and support empirical testing.

### 2.5 Saturation and nonlinear collapse

Buffered systems do not fail gradually. Near saturation, small additional increases in load can precipitate abrupt state transitions. Clinically, this appears as sudden loss of focus, fragmentation of thought, disorganization, or rapid disengagement following a period of intact performance.

This nonlinear collapse explains why engagement in ADHD often fails late in tasks and why disengagement may feel disproportionate to immediate triggers. Collapse reflects saturation of buffering tolerance rather than loss of effort or motivation. Engagement failure may occur even with residual capacity if operational bandwidth is narrow, reflecting failure of ongoing regulation rather than exhaustion.

Empirical support for nonlinear engagement failure in ADHD comes from several converging sources. Time-on-task studies using extended continuous performance paradigms document abrupt increases in response time variability and attentional lapses during the later portions of tasks, a pattern inconsistent with linear fatigue but consistent with threshold-dependent instability (2, 21). Analyses of intra-individual response time distributions using ex-Gaussian modeling reveal that ADHD is characterized by disproportionate increases in the tau (τ) parameter—reflecting the tail of slow, lapsed responses—under sustained demand (24), suggesting threshold-driven rather than gradually degrading performance. In computational psychiatry, analogous threshold-dependent transitions have been modeled as “critical transitions” or “tipping points,” in which systems approaching a stability boundary exhibit early-warning signals such as increasing variance and autocorrelation prior to collapse (26). These models provide a formal precedent for the nonlinear engagement dynamics proposed here and suggest measurable precollapse signatures—specifically, rising intra-individual variability and slower recovery from perturbation—that could be tested empirically in extended sustained-attention paradigms. The concept of buffer capacity employed in the present framework is intentionally positioned at the conceptual rather than neurobiological level: it describes a tolerance threshold governing stability, analogous to a buffering limit in a regulatory system, without presupposing a specific neural substrate. Neuroenergetic correlates—such as dopaminergic signaling in frontostriatal circuits (18)—may ultimately correspond to biological determinants of this threshold, but the present framework does not require this mapping for its predictions to be operationalized or tested.

### 2.6 Translational implications for ADHD

At a formal level, buffering describes lawful system properties, finite tolerance, cumulative load, saturation, and nonlinear collapse—that govern engagement dynamics. Translationally, these properties can be expressed using buffering capacity (BC), buffering load (BL), and buffering reserve (BR = BC − BL), where reserve denotes the remaining margin before instability.

For clinical communication, these interacting properties can be summarized by operational bandwidth (OB): the range of cumulative demand and fluctuation within which engagement remains stable without triggering state transition. Narrow OB yields brittle engagement, whereas wider OB permits tolerance of interruptions, errors, and variability. Clinically, OB is experienced as operational forgiveness—the degree to which the system tolerates imperfection and brief overload without disengaging.

Within this framework, ADHD is characterized not by the absence of executive capacity, but by reduced buffering reserves and chronically narrow operational bandwidth. As a result, sustained engagement becomes highly sensitive to time-on-task, emotional load, interruption, and contextual variability.

This instability biases regulation away from sustained, goal-directed engagement toward reliance on pressure- or salience-driven stabilization. Urgency, novelty, or emotional intensity may transiently stabilize engagement by constraining competing demands, but such stabilization is brittle and collapses once salience dissipates. Inattention and hyperfocus therefore emerge as complementary expressions of the same underlying regulatory instability rather than opposing capacities.

### 2.7 Conceptual formalization of buffering dynamics

At a conceptual level, the buffering framework can be approximated using three interacting variables: cumulative operational load (L), operational capacity (C), and engagement stability (E). Engagement remains stable when L < C, becomes metastable as L approaches C, and may undergo nonlinear collapse when L ≥ C. The rate of load accumulation (dL/dt) is influenced by task demand, emotional load, and contextual factors, while effective capacity may be modulated by pharmacological or environmental supports.

For clarity, the core constructs of the framework are defined as follows. Buffering load (BL) refers to the cumulative cognitive and emotional demand imposed over time. Operational capacity (OC) denotes the maximum cumulative load that can be tolerated while maintaining stable engagement. Buffering reserve (BR) represents the remaining margin before instability (BR = OC − BL). Operational bandwidth (OB) reflects the range of conditions under which engagement remains stable in the presence of perturbation. Demand rate (DR) refers to the rate at which BL accumulates per unit time, modulated by task demands, stress, and emotional load. In the conceptual figures, this tolerance limit is represented as Buffer Capacity (BC), which serves as the schematic expression of Operational Capacity (OC). Thus, BC and OC refer to the same underlying constraint construct, with BC used primarily for conceptual illustration and OC used for clinical and translational discussion.

This formulation is intentionally simplified and does not assume direct measurement of buffering variables. Rather, it provides a conceptual bridge between phenomenological description and empirical testing. Within this framework, observable outcomes—such as time-to-disengagement, intra-individual variability, lapse frequency, and re-engagement latency—serve as behavioral proxies for underlying buffering dynamics. This approach supports the generation of testable predictions while remaining agnostic regarding specific neural implementations.

## 3 Operational economy and pre-volitional state transition

### 3.1 Metastability and constraint-based collapse

Section 2 characterized sustained engagement as a buffered mental state—one that can be maintained under cumulative cognitive and emotional demand if system tolerance is not exceeded. A key implication of this formulation is that engagement does not degrade gradually in a linear fashion. Rather, it operates within a metastable regime: engagement may appear intact yet becomes increasingly sensitive to perturbation as cumulative load approaches tolerance limits. When those limits are exceeded, collapse occurs abruptly.

This pattern contrasts with traditional vigilance or fatigue-based accounts, which assume progressive depletion of effort or control. In the buffered framework, instability emerges not from exhaustion of a resource, but from violation of system constraints. As tolerance is approached, small perturbations—whether cognitive, emotional, or contextual—can precipitate rapid transition. Comparable nonlinear transitions and constraint-driven instabilities have been described across dynamical systems models of cognition and psychopathology (15, 16, 26).

Clinically, this dynamic is familiar. Individuals with ADHD often demonstrate intact performance for a period, followed by sudden shifts in attention, behavior, or affect that appear disproportionate to the immediate situation. These transitions are frequently experienced as involuntary and ego-dystonic. From this perspective, disengagement is not best understood as a failure of motivation or deliberate withdrawal, but as a lawful state transition that occurs when the 

### 3.2 Operational economy as a constraint principle

Operational Economy (OE) refers here to a regulatory constraint framework governing the allocation and stability of engagement under cumulative demand and should not be interpreted as a metabolic or energetic model. Operational Economy describes how mental systems preserve stability while minimizing excessive regulatory costs. As cumulative load increases, buffering reserve narrows and the effort required to sustain engagement rises steeply. Under these conditions, continued regulation becomes inefficient, and the system favors rapid exit over prolonged instability.

Similar cost-minimization principles are well described in biological stress and regulatory systems, where sustained activation beyond tolerance leads to abrupt reconfiguration rather than gradual decline (27, 28). Within this framework, disengagement is not a breakdown of control, but an adaptive response to excessive regulatory demand.

### 3.3 Pre-volitional transition and the global switching framework

A central consequence of this formulation is that disengagement frequently occurs prior to conscious decision-making. Once engagement enters a fragile regime, transition can be triggered by relatively minor perturbations without deliberate intent. Pre-volitional transitions of this kind have been described in studies of cognitive control and large-scale network dynamics, reflecting system-level reconfiguration rather than conscious choice (29–31).

We refer to the functional process mediating this rapid transition as the Global Switching Framework (GSF). GSF does not denote a specific neural circuit, but rather the process by which mental systems reconfigure when stable engagement can no longer be maintained. From this perspective, disengagement represents lawful state transition rather than voluntary withdrawal.

### 3.4 Asymmetry and clinical expression in ADHD

An important feature of this transition is its asymmetry. Disengagement tends to be rapid once buffering constraints are violated, whereas re-engagement requires restoration of those conditions—through reduction of cumulative load, expansion of operational capacity, or recovery of buffering reserve—and therefore proceeds more slowly. Such asymmetric dynamics, including hysteresis and delayed recovery, are well described in nonlinear regulatory systems (26).

Within this framework, ADHD can be understood as a condition in which engagement operates chronically near buffering limits. Reduced reserve and narrow operational bandwidth render the system highly sensitive to cumulative load, such that everyday cognitive or emotional demands rapidly bring engagement into a metastable regime. Under these conditions, Operational Economy favors early disengagement over prolonged unstable regulation. This accounts for the characteristic pattern in ADHD in which engagement is abrupt, context-sensitive, and only weakly correlated with motivation or insight (2, 32).

Over time, regulatory strategies that minimize immediate operational costs—such as urgency-driven activation, salience capture, or rapid disengagement—become recurrent patterns rather than situational responses. This helps explain why ADHD often manifests as cyclical patterns of load accumulation followed by urgent mobilization or collapse, and why purely behavioral interventions rarely produce durable change.

Interventions such as external structure, task simplification, or pharmacological modulation can extend engagement by reducing effective load or increasing tolerance, but they do not abolish the underlying dynamics. Stimulant treatment does not directly “correct” behavior; rather, it reshapes the operational economy by expanding the range of conditions under which sustained engagement can be maintained (22, 33).

### 3.5 Scope and boundaries

**Figure 2:**
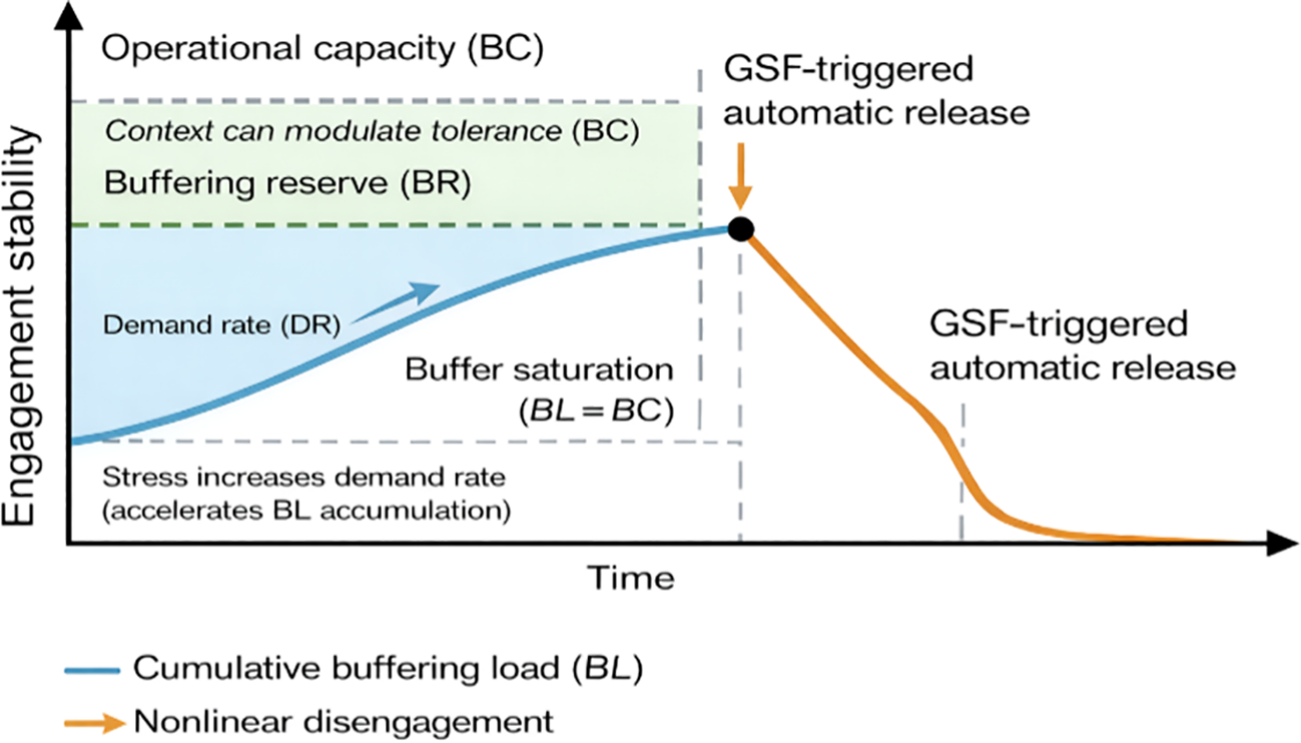
Buffered engagement dynamics and GSF-mediated automatic release under cumulative demand. Sustained mental engagement is depicted as a buffered state whose stability depends on the relationship between cumulative buffering load (BL) and operational capacity (BC). BC = Buffer Capacity: the maximum cumulative load the engagement system can absorb while maintaining stable function; BL = Buffering Load: cumulative cognitive and emotional demand integrated over time; BR = Buffering Reserve: the remaining margin before instability (BR = BC − BL); DR = Demand Rate: the rate at which BL accumulates per unit time, modulated by task demands, stress, and emotional load. As engagement is maintained over time, BL accumulates at a rate determined by task demand and contextual factors (e.g., stress), progressively reducing BR. Engagement remains stable while BL < BC. When cumulative load reaches the tolerance threshold (BL = BC), buffer saturation occurs and a rapid, pre-volitional state transition is triggered via the Global Switching Framework (GSF), resulting in abrupt, nonlinear disengagement. The shaded region labeled ‘Context can modulate tolerance (BC)’ illustrates that environmental and pharmacological factors can expand or contract the tolerance threshold without altering the governing dynamics of the engagement–collapse–release cycle. Operationally, the saturation point corresponds to the moment at which time-to-collapse has been reached; GSF-triggered automatic release represents the transition from engaged to disengaged state, experienced clinically as sudden loss of focus, mental fragmentation, or task abandonment despite continued intent (3). Operational capacity is finite but non-depleting, and disengagement reflects lawful release from an unstable state rather than voluntary withdrawal or gradual fatigue. The figure illustrates constraint-level dynamics governing sustained engagement without specifying neural mechanisms.

These relationships are summarized in **Figure 2**. Sustained engagement is conceptualized as a buffered state whose stability depends on the balance between cumulative load and operational capacity. As load accumulates, buffering reserve narrows until a threshold is reached, at which point rapid state transition occurs. Importantly, operational capacity is finite but not progressively depleted, and disengagement reflects lawful release from an unstable state rather than gradual fatigue.

## 4 The global switching framework and mental metastability

### 4.1 GSF as a pre-volitional state-transition mechanism

A buffered system requires not only finite capacity and load absorption, but also a mechanism for resolving instability once tolerance limits are exceeded. Without such a mechanism, systems would remain trapped in fragile configurations or drift into prolonged degradation without clear state resolution. In mental regulation, sustained engagement cannot remain stable once cumulative cognitive and emotional demand exceeds operational capacity. A rapid state transition is therefore required to restore stability.

Within the present framework, this role is fulfilled by the Global Switching Framework (GSF). Operational Economy specifies the conditions under which engagement becomes metastable and unstable; GSF specifies how this instability is resolved through automatic state transition. GSF does not represent executive control, motivation, or conscious choice. Rather, it denotes the pre-volitional process by which engagement exits an unsustainable configuration once buffering constraints are violated.

### 4.2 Pre-volitional transition and system-level reconfiguration

GSF operates prior to conscious decision-making and is largely independent of explicit intention. As cumulative operational load approaches tolerance limits, engagement enters a fragile regime in which small perturbations can precipitate abrupt disengagement. Subjectively, this transition is experienced as involuntary and ego-dystonic—attention fragments, effort collapses, or the task is abandoned despite ongoing intent (3).

These transitions reflect system-level reconfiguration rather than conscious decision-making. Comparable pre-volitional shifts and large-scale network reorganization have been described across cognitive and affective domains without requiring explicit awareness or voluntary control (29–31). From this perspective, disengagement represents a lawful state transition governed by system constraints rather than a failure of will or intention.

Importantly, GSF does not reflect regulatory failure. It functions as a protective mechanism, preventing prolonged instability and excessive internal strain once engagement can no longer be sustained.

### 4.3 Metastability, hysteresis, and asymmetric engagement dynamics

Building on Section 3, metastability here refers to the conditions under which GSF-mediated transitions are triggered. Sustained engagement remains stable under moderate cumulative demand yet becomes increasingly sensitive to perturbation near tolerance limits. Once these limits are exceeded, collapse occurs nonlinearly rather than gradually, a pattern inconsistent with linear fatigue or depletion models (2, 21).

Operational Economy therefore predicts asymmetry between disengagement and re-engagement. Disengagement is rapid once instability is detected, whereas re-engagement requires restoration of buffering conditions—through reduction of cumulative load, widening of tolerance, or recovery of reserve—and proceeds more slowly. Such hysteresis-like dynamics, including delayed recovery following collapse, are well described in nonlinear regulatory systems (26).

### 4.4 GSF and clinical expression in ADHD

In ADHD, engagement operates chronically near the limits of buffering tolerance, with reduced reserve and narrow operational bandwidth. As a result, metastable regimes are entered more frequently, and GSF-mediated release is triggered under levels of cumulative demand that would not disrupt engagement in individuals with greater buffering capacity.

Clinically, this produces recurrent cycles of intact initiation followed by abrupt disengagement and delayed re-entry—patterns that are lawful and demand-conditional rather than motivational or oppositional (2, 32). Because insight and intention are preserved, these cycles are often experienced as personally inexplicable. Over time, repeated disengagement despite effort may be misinterpreted as moral or character failure, contributing to chronic self-blame, shame, and guilt.

Within this framework, the Global Switching Framework itself need not be impaired in ADHD. The primary alteration lies upstream, in constrained buffering capacity. As a result, GSF is activated more frequently and at lower thresholds of cumulative load. Under low-salience conditions, this manifests as distractibility or task abandonment. Under high-salience or urgent conditions, engagement may become transiently over-stabilized, producing pressure-driven lock-in. In both cases, the same underlying mechanism governs the resolution of engagement instability.

Importantly, GSF introduces no additional explanatory burden beyond the buffering constraints defined by Operational Economy. Its role is to clarify the timing, phenomenology, and asymmetry of disengagement as a lawful state transition, preserving parsimony while improving explanatory precision. GSF is not proposed as a discrete anatomical network, but as a functional class of rapid state transitions consistent with known large-scale reconfigurations observed during loss of cognitive stability (15, 16, 31).

## 5 Symptom clustering and behavioral paradoxes in ADHD

**Table 1** summarizes the clinical expression of ADHD symptoms in terms of operational capacity, buffering dynamics, and metastability. This section applies the buffering and state-transition framework developed in Sections 2–4 to core ADHD phenomenology. The aim is integrative: to show how inattention, impulsiveness, hyperactivity, hyperfocus, and disorganization arise from a single constraint on sustained engagement under cumulative demand, rather than from multiple independent deficits.

**Table 1:** summarizes the clinical expression of ADHD symptoms in terms of operational capacity, buffering dynamics, and metastability.

Symptom cluster	Core regulatory issue	Operational interpretation (OC/OM)	Typical clinical presentation
Inattention	Failure to stabilize engagement under low salience	Reduced buffering reserve; engagement becomes metastable early	Careless mistakes, drifting attention, premature disengagement during routine tasks
Impulsivity	Rapid state exit under instability	Narrow operational bandwidth favors fast switching to reduce cost	Acting before thinking, interrupting, abandoning tasks abruptly
Hyperactivity	Motor discharge under regulatory strain	Engagement instability expressed somatically	Fidgeting, restlessness, difficulty remaining still during sustained demand
Hyperfocus	Salience-driven over-stabilization	Temporary fixation with reduced switching flexibility	Prolonged focus under urgency or novelty with difficulty disengaging
Disorganization/procrastination	Cumulative load with delayed re-engagement	Anticipated buffer saturation leads to avoidance or collapse	Task pile-up, last-minute mobilization, repeated start–stop cycles

### 5.1 Engagement instability: inattention, variability, and hyperfocus

Inattention in ADHD is most prominent in low-salience, routine, or weakly structured contexts. Within the present framework, this reflects failure to stabilize engagement over time rather than absence of attentional capacity or effort. As engagement approaches its operational limits, it becomes increasingly sensitive to perturbation, resulting in drifting attention, missed details, and premature disengagement despite intact understanding and intention. Time-on-task instability under otherwise constant task conditions is a consistent feature of ADHD (2, 32).

Marked variability—poor persistence in routine contexts alongside intense focus under urgency or novelty—is a central ADHD paradox. Rather than reflecting fluctuating ability, this pattern reflects context-dependent stabilization of engagement. Under high salience or immediate consequence, competing demands are constrained and effective load is reduced, allowing engagement to stabilize transiently.

Hyperfocus therefore represents salience-driven over-stabilization of engagement, characterized by narrowed attentional allocation, reduced flexibility, and impaired disengagement. This interpretation is consistent with findings linking hyperfocus to attentional capture and reward salience (34), in which highly engaging stimuli transiently reduce effective buffering load by constraining internal competition. The over-stabilization of engagement under high-salience conditions draws specifically on mechanisms of bottom-up attentional capture—when motivationally significant stimuli are present, exogenous orienting effectively suppresses competing demands and reduces the functional demand rate (35), thereby extending the window of stable engagement without increasing operational capacity. Hyperfocus does not indicate superior capacity. When salience diminishes, engagement again becomes unstable and disengagement recurs. Inattention and hyperfocus are thus complementary expressions of the same underlying instability rather than opposing phenomena.

**Figure 3:**
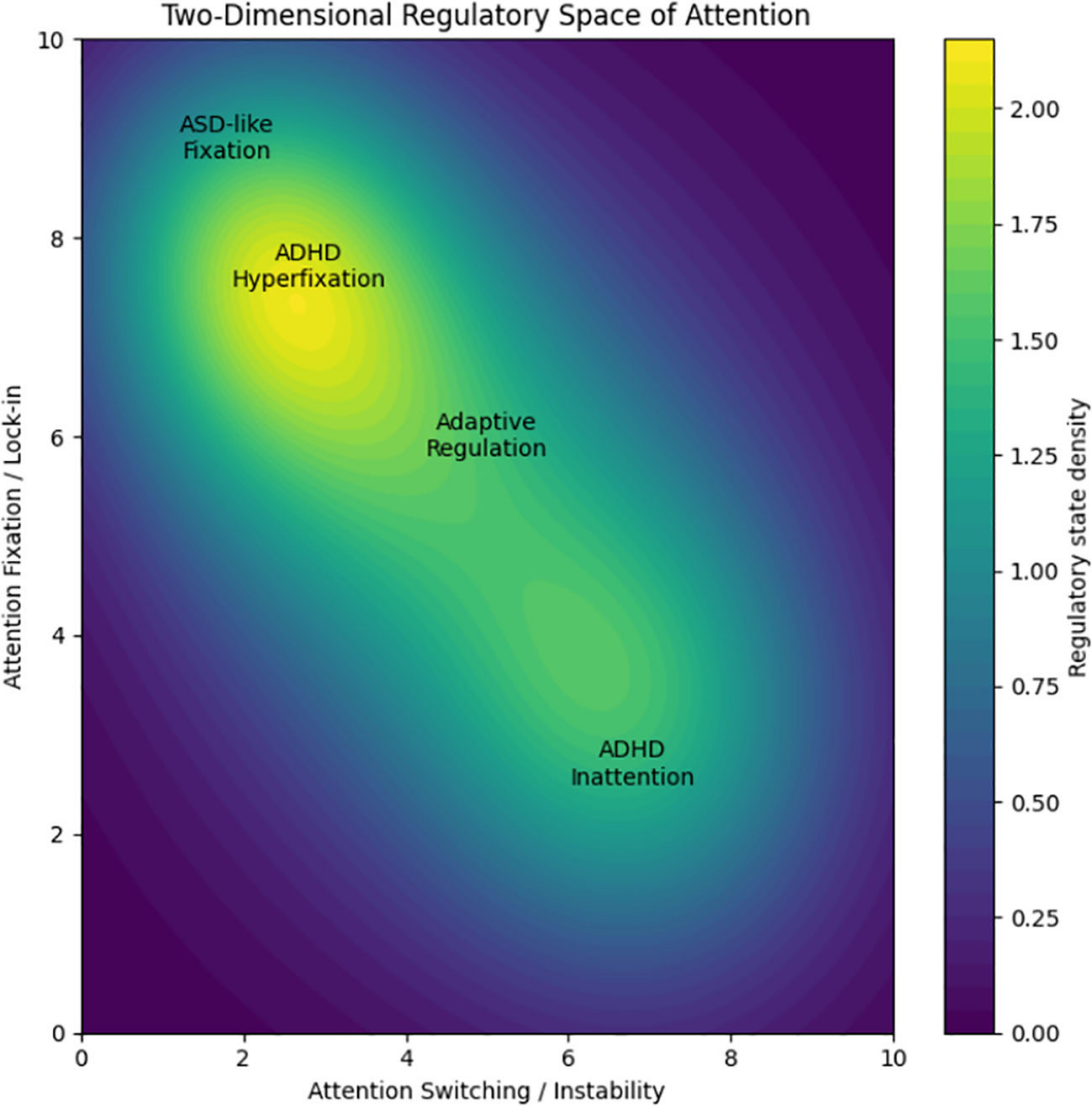
Two-dimensional regulatory space of attention. The figure depicts attentional regulation as a continuous space defined by two dimensions: Attention Switching/Instability (x-axis) and Attention Fixation/Lock-in (y-axis). Color intensity (regulatory state density) identifies modal attentional states. ADHD inattention occupies a region of high switching/instability with low fixation. ADHD hyperfixation occupies a region of high fixation with moderate switching instability. Adaptive regulation occupies an intermediate zone of balanced stability and flexibility. ASD-like fixation occupies the high-fixation, low-instability quadrant. The framework predicts that ADHD is characterized by instability in the regulation of the fixation–switching balance, rather than a fixed position in this space.

These dynamics are illustrated in **Figure 3**, which conceptualizes attentional regulation as a continuous space defined by engagement stability and switching flexibility.

### 5.2 Rapid transitions and cumulative load: impulsivity and disorganization

Impulsivity and hyperactivity reflect rapid state transitions when stable engagement can no longer be maintained economically. As regulatory demands increase, the cost of sustaining controlled engagement rises steeply, favoring fast exits or shifts that reduce immediate strain. Behaviorally, this appears as interrupting, acting prematurely, or motor restlessness—not because inhibitory knowledge is absent, but because sustained regulation becomes inefficient under instability.

Disorganization and procrastination arise from repeated cycles of engagement collapse and delayed re-engagement. Tasks requiring prolonged sequencing, ambiguity tolerance, or sustained planning impose cumulative load that is difficult to maintain within available buffering capacity. When anticipated demands exceed tolerance, disengagement may occur prospectively rather than reactively.

Within this framework, procrastination reflects anticipatory protection against instability, compounded by reduced capacity to sustain engagement across initiation, maintenance, switching, and completion (36). Over time, accumulation-and-emergency-discharge cycles dominate regulation, producing chronic disorganization and last-minute mobilization.

### 5.3 Language organization and sustained meaning integration

Language production places sustained demands on integration, sequencing, monitoring, inhibition of tangential associations, and emotional regulation. Individuals with ADHD often demonstrate preserved local coherence alongside impaired global organization: sentences remain grammatically correct and meaningful, yet discourse drifts or fragments over time (32).

Within the buffering framework, this pattern reflects failure of sustained meaning integration under cumulative demand rather than deficits in linguistic knowledge or representation. As engagement becomes unstable, global coherence deteriorates while local processing remains intact. Tangentiality therefore reflects instability of sustained integration rather than primary language impairment.

This account explains why language organization worsens with prolonged conversation, emotional load, or fatigue and improves in short, structured, or highly scaffolded exchanges.

### 5.4 Symptom clustering and domain-specific vulnerability

ADHD symptoms cluster preferentially in domains that impose high sustained demands such as planning, writing, organization, emotional regulation, and extended social interaction (1). This clustering follows directly from task–capacity mismatch: domains with higher demand rates drive engagement more rapidly toward instability.

This perspective explains symptom clustering and apparent comorbidity without invoking multiple independent deficits or domain-specific dysfunction. Diverse behavioral expressions arise from a shared constraint on sustained engagement under cumulative demand.

Although the present analysis focuses on ADHD, similar demand-dependent instability may contribute to symptoms in overlapping conditions such as anxiety or depression, where emotional load accelerates accumulation and destabilization (8). This suggests a broader applicability of the buffering framework across psychiatric conditions characterized by impaired sustained engagement.

### 5.5 Affective dysregulation and emotional load in ADHD

Emotional dysregulation is among the most clinically significant and functionally impairing features of ADHD, yet it remains underrepresented in theoretical accounts that emphasize cognitive and executive processes (6, 8). Within the present framework, emotional perturbations are not treated as a separate domain but as a direct and potent contributor to cumulative buffering demand, with important implications for understanding affective instability in ADHD.

Emotional stimuli—whether internal (anticipatory anxiety, frustration, shame, rejection distress) or external (interpersonal conflict, perceived criticism, social evaluation)—accelerate the rate of load accumulation (dBL/dt), thereby shortening the interval before engagement approaches the tolerance threshold. In this formulation, emotions function primarily as load accelerators: they do not alter operational capacity or modify the threshold at which the Global Switching Framework is triggered, but they increase the speed at which cumulative demand builds, effectively compressing the time available for sustained engagement under emotionally laden conditions. This mechanism accounts for the well-documented finding that ADHD symptoms worsen substantially in emotionally charged contexts, independent of motivational factors.

Under conditions of intense emotional perturbation, emotional load may additionally function as a switching trigger: when an emotional event produces a rapid spike in effective demand, it can precipitate GSF-mediated disengagement directly, even when prior cumulative load is relatively low. This accounts for the disproportionate, context-sensitive emotional responses characteristic of ADHD—including rejection sensitive dysphoria (6, 37), affective lability, and abrupt emotional disengagement—which can appear out of proportion to the precipitating stimulus but are interpretable as GSF-triggered transitions in a system operating chronically near its tolerance boundary.

Emotional dysregulation in ADHD therefore reflects a dynamic amplification of engagement instability rather than an independent regulatory system. Literature on emotional lability in ADHD (8) and rejection sensitive dysphoria (37) documents rapid, intense emotional responses that resolve quickly once the triggering context changes—a pattern consistent with rapid GSF-mediated state transitions and subsequent recovery of buffering reserve once demand diminishes. These features are distinct from mood disorders, in which affective perturbations reflect endogenous state changes rather than load-triggered transitions.

Importantly, the present framework predicts that emotional load and cognitive load interact multiplicatively rather than additively in determining time-to-disengagement in ADHD. Specifically, individuals with ADHD who face both high cognitive demand and elevated emotional load should show disproportionately accelerated engagement collapse relative to controls and relative to conditions of elevated cognitive or emotional load alone. This prediction distinguishes the OE/GSF account from motivational models, which would attribute emotional effects primarily to incentive-based engagement modulation, and from executive-deficit models, which treat emotion and cognition as modular systems with largely independent contributions to impairment.

## 6 Pharmacological and contextual modulation of engagement buffering

This section situates pharmacological and contextual treatment effects within the buffering framework developed in Sections 2–4. Interventions are conceptualized not as repairing executive deficits or altering regulatory mechanisms, but as modulating the conditions under which sustained engagement can be maintained. Specifically, treatments influence engagement by shifting the relationship between cumulative demand and operational tolerance, thereby extending the duration over which engagement remains stable without altering the governing dynamics defined by Operational Economy or the automatic release process implemented by the Global Switching Framework (GSF).

### 6.1 Treatment as modulation of buffering conditions

Within this framework, treatment effects are best understood as changes in buffering conditions rather than changes in control architecture. Interventions alter either the system’s tolerance to cumulative demand or the rate at which demand accumulates, thereby widening the margin within which engagement can be sustained.

This formulation is used as a translational lens rather than a mechanistic claim. It does not posit new neural processes or computational operations, nor does it assume direct measurement of buffering variables. Instead, it provides a unified clinical language for understanding how diverse interventions stabilize engagement while preserving the underlying engagement–collapse–release dynamics described earlier.

### 6.2 Dopaminergic modulation and engagement stability

Dopamine-enhancing treatments reliably improve sustained engagement in ADHD, manifesting clinically as prolonged time-on-task, reduced variability, and delayed disengagement under cumulative demand (33, 38). Within the present framework, these effects are most parsimoniously interpreted as modulation of engagement buffering conditions rather than enhancement of executive knowledge or motivation.

Stimulant effects can be understood as increasing tolerance to cumulative demand while simultaneously reducing interference from task-irrelevant internal activity. In functional terms, this improves the signal-to-noise ratio of ongoing cognition, allowing engagement to remain stable for longer periods without increasing peak performance capacity. By slowing the effective accumulation of cognitive and emotional strain, stimulants extend the window over which engagement remains viable.

This interpretation is consistent with evidence implicating frontostriatal systems in sustained engagement stability, which show structural and functional alterations in ADHD (39). Importantly, dopaminergic modulation does not alter the engagement–disengagement dynamics or the automatic release process described in Sections 3–4. Instead, it shifts the operating range within which those dynamics unfold, delaying saturation without changing the governing law of collapse.

Crucially, stimulant effects are most evident in time-dependent measures—such as reduced variability, fewer late-stage lapses, and improved vigilance—rather than in brief or peak executive-function performance. This dissociation is consistently observed in ADHD pharmacology and is directly captured by the buffering framework. **Table 2** summarizes the clinical interpretation of stimulant effects on engagement dynamics, distinguishing improvements in endurance, stability, and tolerance from relatively modest effects on peak performance.

**Table 2:** Core constructs of the operational-buffering framework and their clinical interpretation, including the interpreted effects of stimulant medication on engagement dynamics.

Construct	Clinical meaning	Typical presentation in ADHD	Effect of stimulants (clinical interpretation)
Operational Capacity (OC)	Total cumulative mental demand that can be tolerated before engagement regulation fails	Can function well in short bursts or structured tasks but cannot sustain demands across the day	Slight–moderate ↑ (patients report being able to “handle more” before feeling overwhelmed)
Operational Load (OL)	Rate of cumulative cognitive and emotional strain during ongoing activity	Load accumulates rapidly from distraction, emotion, task-switching, and internal tension	↓ (reduced distractibility and emotional interference slow load accumulation)
Operational Bandwidth (OB)	Usable margin for stable engagement at a given moment	Narrow margin for error; small interruptions or added demands precipitate collapse	Moderate ↑ (greater tolerance for interruptions, errors, and complexity)
Operational Metastability (OM)	Stability of engagement as tolerance limits are approached	Abrupt disengagement, emotional blow-off, or impulsive shifts under pressure	Stabilized toward sustained engagement (less brittle collapse, smoother continuation)
Executive Function (peak)	Maximum performance on brief, structured tasks	Often intact at baseline	≈ unchanged
Executive endurance	Sustained executive performance over time (time-on-task)	Rapid decline with time-on-task	↑↑ (robust extension of time-on-task)

### 6.3 Contextual modulation: stress, structure, and load dynamics

Contextual factors strongly influence the rate at which cumulative demand develops over time. Stress, emotional tension, ambiguity, and environmental chaos accelerate the accumulation of demand, while external structure, task simplification, pacing, and emotional containment reduce it (23).

This distinction explains the pronounced context sensitivity of ADHD. Under high-load conditions, engagement becomes unstable more rapidly, whereas structured and predictable environments allow engagement to be sustained for longer periods without altering underlying capacity. Importantly, these contextual effects do not eliminate instability. They shift the conditions under which instability emerges, extending the duration of stable engagement without changing its governing dynamics.

### 6.4 Empirical grounding and clinical implications

A large body of empirical work examining time-on-task effects in ADHD aligns closely with the buffered engagement framework. Across experimental paradigms, ADHD is characterized not by uniformly impaired initial performance, but by progressive instability under sustained demand. These patterns are consistent with a broader literature on intra-individual variability and late-emerging lapses in ADHD, which are more pronounced under sustained task demands.

Sustained attention and vigilance studies consistently show that performance deteriorates disproportionately over time relative to controls, with increasing variability and late-emerging lapses rather than steady linear decline (2, 21). Early task performance is often intact, diverging only as engagement is maintained, consistently with preserved capacity but reduced tolerance for cumulative demand. Within the buffering framework, this pattern is interpreted as a progressive approach toward capacity limits, in which engagement remains stable until buffering reserve is sufficiently reduced, after which instability becomes increasingly likely.

Motor and cognitive control studies further demonstrate that engagement often fails abruptly after periods of apparent stability rather than degrading gradually. Such nonlinear collapse patterns are not readily explained by models based on gradual fatigue or depletion, but are consistent with systems approaching a stability boundary, in which small additional load can trigger rapid state transition.

Naturalistic observations echo this pattern. Individuals with ADHD may remain engaged for variable intervals before disengaging suddenly, often without clear external provocation (40). Divergence from controls increases with task duration and cumulative demand, reinforcing the central role of time-dependent instability.

Beyond attentional lapses, sustained demand also gives rise to characteristic compensatory behaviors. As engagement becomes fragile, individuals may increase monitoring, checking, rigidity, or over-control to prevent errors or loss of control. These behaviors can resemble obsessive–compulsive symptoms but are context-dependent and emerge under sustained demand rather than from primary obsessional processes. Within this framework, they reflect compensatory responses to narrow operational bandwidth and metastable engagement rather than independent compulsive pathology. These patterns are summarized in **Table 3**.

**Table 3:** Differentiating ADHD-related compensatory OCD-like control behaviors from primary obsessive–compulsive disorder.

Dimension	ADHD with compensatory OCD-like control	Primary OCD
Core driver	Engagement instability and error vulnerability under cumulative demand	Intrusive obsessions with anxiety-driven compulsions
Relationship to attention	Secondary to inattention and careless errors	Independence of attentional instability
Function of rigidity	Compensatory attempts to prevent mistakes or disengagement	Pathological ritualization to relieve obsessional anxiety
Flexibility	Context-dependent; improves when engagement stabilizes	Rigid and context-insensitive
Insight	Preserved; behavior experienced as effortful but necessary	Variable; often reduced
Temporal pattern	Emerges under pressure, salience, or sustained demand	Persistent across contexts
Relationship with engagement buffering	Reflects narrow operational bandwidth and fragile metastability	Not primarily related to engagement buffering
Effect of stimulants	May transiently intensify compensatory rigidity by enabling endurance under high demand, or reduce rigidity when engagement stability improves sufficiently	Minimal or inconsistent benefits; may worsen anxiety
Clinical trajectory	Improves as engagement endurance and tolerance increase	Chronic unless OCD-specific treatment is applied
Diagnostic implication	Secondary compensatory behavior, not true OCD	Primary obsessive–compulsive disorder

Taken together, these findings converge on three features central to the present framework: sensitivity to cumulative demand, metastable engagement, and nonlinear disengagement. Within this formulation, performance variability and late-emerging breakdown are interpreted as signatures of a system approaching its stability boundary under sustained load.

These findings are consistent with state-regulation accounts of ADHD but also suggest that time-dependent instability may not be fully captured by continuous models of arousal or effort. In particular, the emergence of late-stage performance breakdown, increased variability, and abrupt lapses under sustained demand aligns with a framework in which engagement stability is bounded and subject to threshold-dependent transition (e.g., 2). This supports the need for models that explicitly incorporate cumulative load and stability limits in explaining sustained engagement failure.

## 7 Discussion

### 7.1 Reframing ADHD as a disorder of operational capacity

This article reframes ADHD not as a disorder of executive ability, motivation, or intelligence, but as a disorder of operational capacity for sustained engagement. Rather than emphasizing what individuals with ADHD can do at a given moment, the present framework centers on how long engagement can be stably maintained under cumulative cognitive and emotional demand.

By conceptualizing engagement as a buffered state governed by Operational Economy, this account explains why individuals with ADHD may demonstrate intact understanding and initial performance yet disengage reliably over time. This reframing aligns closely with everyday clinical phenomenology and resolves longstanding paradoxes—such as preserved insight alongside repeated failure of follow-through—that remain difficult to reconcile within dominant explanatory models.

### 7.2 Relationship to existing models

Executive-function models have provided valuable insights by identifying impairments in inhibition, working memory, planning, and cognitive flexibility in ADHD (6, 41). These models are particularly informative for explaining performance on brief or highly structured tasks. However, they primarily characterize static abilities or trait-level regulation and do not formally account for time-dependent instability, nonlinear collapse, and re-engagement asymmetry—features that are central to ADHD in everyday contexts (2, 21).

As outlined in Sections 3–4, the buffering framework complements rather than replaces executive-function models by clarifying the conditions under which executive processes fail. Executive knowledge and skill may remain intact yet become functionally ineffective when engagement cannot be sustained under cumulative demand. In this view, executive dysfunction emerges as a downstream consequence of engagement instability rather than a primary deficit.

The present framework also differs from depletion- or fatigue-based accounts. Rather than positing gradual exhaustion of mental resources, it describes lawful instability in a buffered system, in which cumulative demand leads to threshold-dependent collapse. This distinction explains why engagement failure in ADHD is often abrupt, disproportionate to immediate triggers, and difficult to reverse—patterns that are not well captured by linear fatigue models.

### 7.3 Differential diagnosis and longitudinal trajectories

A central strength of the buffering framework lies in its ability to distinguish ADHD from other psychiatric conditions that share superficial behavioral features. Comparison with executive dementias illustrates this point. These distinctions are summarized in **Table 4**.

**Table 4:** Differentiating ADHD from executive dementias (e.g., behavioral-variant frontotemporal dementia) within the buffering framework.

Dimension	ADHD	Executive dementia (e.g., bvFTD)
Primary pathology	Temporal instability of regulation	Structural degradation of executive systems
Executive knowledge	Preserved	Lost or degraded
Operational Capacity (OC)	Present but constrained	Progressively reduced
Operational Metastability (OM)	Imprecise/poorly tuned	Often rigid or globally degraded
Time-on-task effect	Abrupt collapse after initial stability	Early and persistent impairment
Disorganization	State-dependent (maintenance failure)	Trait-like (capacity loss)
Response to urgency	Improves performance	Often worsens confusion
Regulation mode	Pressure-/salience-driven	Weak or absent goal-driven control
Reversibility	Context- and medication-sensitive	Limited, progressive
Phenomenology	Ego-dystonic, frustrating	Often anosognosic
Medication effect	Extends engagement endurance	Minimal or symptomatic only

A similar distinction applies to bipolar disorder. ADHD may present with episodic over-engagement, affective intensity, and behavioral disinhibition, creating the appearance of mania. However, longitudinal evidence indicates that individuals with ADHD rarely develop true bipolar disorder despite frequent diagnostic confusion (42). Within the present framework, such “mania-like” states reflect emergency discharge of accumulated demand under conditions of saturation rather than endogenous mood-state transitions. These episodes are typically context-dependent, insight-preserved, and reversible, in contrast to the autonomous and phase-based nature of bipolar mood episodes.

In contrast, ADHD is associated with a substantially elevated risk of major depressive disorder across development (1, 43). From the buffering perspective, depression emerges when repeated cycles of accumulation and discharge lose compensatory effectiveness, resulting in sustained negative affect, demoralization, and reduced engagement capacity. These distinct cycling dynamics are summarized in **Table 5**.

**Table 5:** Differentiating ADHD from bipolar disorder: cumulative-load cycling dynamics versus endogenous mood-state transitions.

Dimension	ADHD	Bipolar disorder
Core driver	Cumulative operational load	Endogenous mood state
Cycling trigger	Load saturation → emergency discharge	Mood episode onset
Regulation mode	Pressure/salience-driven	Mood-congruent
Temporal pattern	Irregular, context-dependent	Episodic, phase-based
Insight during episodes	Preserved	Often reduced
Response to structure	Improves stability	Limited
Medication effect	Extends endurance	Mood stabilization
Reversibility	High load reduction	Variable, often delayed

A related but distinct pattern involves the emergence of OCD-like control behaviors in some individuals with ADHD. As discussed in Section 6, these behaviors are best understood as compensatory responses to chronic engagement instability rather than primary obsessive–compulsive disorder. They are context-dependent, load-sensitive, and fluctuate with operational stress, reflecting attempts to externally stabilize engagement under constrained buffering conditions (see **Table 3**). Recognizing this pattern helps differentiate adaptive but costly regulation from primary pathology.

### 7.4 Mechanism and clinical implications

A central implication of this framework is that engagement failure frequently occurs prior to conscious choice. As detailed in Sections 3–4, disengagement reflects automatic release when tolerance limits are exceeded, rather than voluntary withdrawal or insufficient willpower. The Global Switching Framework (GSF) provides a conceptual account of how such transitions are implemented, enabling rapid reconfiguration of mental state once stable engagement can no longer be maintained.

Clinically, this perspective offers a unified account of symptom clustering, context sensitivity, and partial treatment response in ADHD. It helps explain why symptoms worsen under stress, why urgency and external structure can transiently stabilize functioning, and why pharmacological and environmental interventions may act through distinct but complementary pathways (1).

Importantly, the translational constructs of buffering capacity, cumulative demand, and engagement stability provide clinicians with an intuitive language for discussing these dynamics with patients. By shifting the focus from effort and motivation to constraint and stability, this framework reduces moralized interpretations of disengagement such as laziness or lack of discipline, supporting a more accurate and humane understanding of the disorder.

The present framework is not intended as a complete mechanistic account but as a constraint-level hypothesis that organizes clinical phenomenology and generates testable predictions. Consistent with this view, real-world studies using ecological momentary assessment indicate that attentional engagement in ADHD fluctuates markedly across contexts and time, supporting the interpretation that instability of sustained engagement, rather than fixed deficit, contributes to functional impairment.

From a clinical perspective, capacity-focused interventions may include pacing strategies that limit cumulative load, structured scaffolding of tasks to reduce demand rate, and load-distribution approaches that intersperse high-demand periods with recovery intervals. These strategies aim to extend stable engagement windows by modifying load dynamics rather than enhancing momentary control. Concrete examples include: breaking complex tasks into shorter segments (e.g., 20–25 minute work blocks with structured breaks) to prevent progressive buffer depletion; using external prompts or deadlines to regulate demand rate and prevent uncontrolled load accumulation; incorporating scheduled pauses to allow partial buffering reserve recovery before saturation is reached; and reducing environmental emotionally loaded stimuli to lower the affective contribution to demand rate. These capacity-focused strategies are distinct from purely skill-based interventions in that they aim to modulate the load side of the engagement equation rather than directly training executive functions.

### 7.5 Empirical considerations and scope

The present framework is intentionally formulated at the level of functional constraints and clinical phenomenology. Current neuroimaging and electrophysiological tools lack sufficient temporal resolution, ecological validity, and reliability to serve as direct clinical indices of engagement buffering (22). This limitation reflects the current state of measurement rather than the weakness of the conceptual model.

Future work should focus on time-sensitive behavioral measures capable of capturing engagement stability, threshold dynamics, and re-engagement latency. Extended continuous performance tasks, immersive task environments, and ecological momentary assessment offer promising avenues for operationalizing these constructs in both research and clinical settings.

Operational Economy is proposed as a general principle of sustained engagement rather than a mechanism unique to ADHD. ADHD is distinguished by a chronically reduced margin between operational capacity and everyday task demands, rendering engagement instability frequent and clinically salient. While similar constraint dynamics may contribute to symptoms in other conditions, the present framework maintains clear boundaries by assuming preserved self-integrative buffering in ADHD, in contrast to disorders such as schizophrenia.

### 7.6 Integrative summary

Taken together, these findings position ADHD as a disorder of sustained engagement governed by lawful buffering constraints rather than deficits in executive knowledge or motivation. Operational Economy defines the limits of engagement stability, the Global Switching Framework accounts for automatic state transitions, and buffering constructs provide a bridge between theoretical principles and lived clinical experience.

This integrative perspective reframes ADHD in a manner that is coherent, clinically meaningful, and closely aligned with everyday psychiatric practice, offering a unified account of its phenomenology, treatment response, and longitudinal trajectory.

## 8 Predictions, falsification, and empirical implications

A central strength of the present framework is that it generates specific, falsifiable predictions regarding engagement stability, demand sensitivity, and state transition dynamics in ADHD. These predictions are designed to distinguish the buffering framework from models based on continuous modulation of arousal, effort, or structural capacity loss. They can be tested using established behavioral paradigms, physiological measures, and experimental manipulations of task structure and demand, and include explicit conditions under which the framework would be invalidated.

To support empirical testing, these predictions can be operationalized using established paradigms such as Continuous Performance Tasks (CPT), virtual classroom environments, and ecological momentary assessment (EMA) approaches capable of capturing dwelling time, collapse thresholds, and re-engagement asymmetry in both laboratory and real-world settings.

### 8.1 Time-on-task effects

In ADHD, engagement stability is predicted to deteriorate as a function of cumulative time-on-task even when task demands and incentives are controlled. Extended CPT paradigms are expected to reveal initially intact performance followed by abrupt increases in variability and late-emerging attentional lapses, resulting in shorter and more variable dwelling times than controls (2).

This pattern contrasts with executive dementias, where impairment is present early and relatively insensitive to task duration, and with bipolar disorder, where performance fluctuations are not systematically linked to cumulative demand. A critical falsification condition would be the demonstration that individuals with ADHD can maintain stable engagement over extended durations once initial performance is matched, independent of cumulative time-on-task. Such findings would challenge the buffering formulation.

These predictions can be tested using extended-duration CPTs (20–30 minutes) with time-resolved analyses of variability (e.g., ex-Gaussian parameters, lapse clustering) to detect nonlinear collapse rather than gradual decline.

### 8.2 Demand-rate manipulation

Increasing demand rate through sustained integration requirements, ambiguity, multitasking, or emotional load is predicted to shorten dwelling time disproportionately in ADHD, even when total task duration and motivational incentives are controlled. In virtual classroom paradigms, the addition of distractors or emotionally salient stimuli is expected to accelerate engagement collapse relative to low-demand conditions.

This sensitivity to demand rate distinguishes ADHD from conditions in which engagement failure reflects absolute capacity loss rather than cumulative load. A key falsification condition would be evidence that engagement failure depends primarily on task difficulty or reward valuation, rather than on cumulative demand and the rate of load accumulation.

Empirical testing can be implemented using factorial designs that manipulate demand rate while holding total duration constant, with time-to-disengagement measured via behavioral markers, eye tracking, or structured interruption reports. These predictions are consistent with prior findings demonstrating sensitivity to event rate and temporal structure in ADHD, as well as with physiological indices of arousal such as pupil dilation, which track dynamic engagement under varying demand conditions (19, 20).

### 8.3 Stress and emotional load interactions

Stress and emotional tension are predicted to accelerate engagement collapse in ADHD by increasing buffering load, leading to earlier disengagement without changes in task comprehension or incentive value. Mild stress induction—such as time pressure, evaluative context, or environmental noise—is expected to reduce dwelling time more markedly in ADHD than in controls.

This load-dependent effect contrasts with bipolar disorder, where stress may precipitate mood episodes that are not systematically tied to task engagement dynamics. The buffering account would be challenged if stress effects could be fully explained by distraction or motivational withdrawal alone, without measurable changes in time-to-collapse or engagement stability.

Ecological momentary assessment (EMA) studies further demonstrate substantial within-day fluctuations in attention, thought content, and engagement in individuals with ADHD, providing real-world evidence of unstable engagement states and rapid shifts in cognitive focus. These patterns are consistent with the proposed framework, in which stress increases cumulative load and accelerates transitions from stable engagement to disengagement.

Within the present framework, emotional load is not treated as a separate domain but as a direct contributor to cumulative buffering demand. Emotional salience, stress, and affective reactivity therefore modulate both the rate of load accumulation and the proximity to stability boundaries. In this way, emotional dysregulation in ADHD can be understood as a dynamic amplification of engagement instability, rather than as an independent process operating outside the engagement system. Specifically, the framework predicts that conditions associated with rejection sensitivity or emotional salience will produce disproportionate acceleration of engagement collapse in ADHD relative to controls, and that this effect will be attenuated by pharmacological treatment in proportion to its effects on operational load accumulation rate.

### 8.4 Pharmacological modulation

Dopamine-enhancing treatments are predicted to extend dwelling time and delay engagement collapse in ADHD without normalizing peak executive performance or eliminating variability. Within the buffering framework, pharmacological effects shift tolerance limits rather than abolish engagement–disengagement dynamics (33, 38).

This pattern distinguishes OE/GSF predictions from both motivational and executive models. Motivational accounts would predict that stimulants primarily improve performance on tasks with sufficient reward value, without necessarily extending time-to-collapse on low-incentive sustained tasks. Executive-function models would predict that stimulants should improve peak performance on brief standardized tasks, with time-dependent effects being secondary. The OE/GSF framework specifically predicts the opposite dissociation: robust extension of engagement duration and reduction of late-stage lapses with relatively modest improvements in peak accuracy—a dissociation that is well-documented in the clinical pharmacology of stimulants (38, 44).

A critical falsification condition would be evidence that stimulant treatment eliminates time-dependent instability or primarily improves peak performance rather than extending tolerance for sustained engagement. These effects can be tested using double-blind crossover designs comparing on- and off-medication performance in sustained-attention tasks, with analyses focused on time-to-collapse and variability rather than maximal accuracy.

### 8.5 Distinction from executive-function models

Measures sensitive to engagement duration, re-engagement difficulty, and collapse dynamics are predicted to better account for functional impairment and treatment response in ADHD than traditional executive-function scores.

If static executive-function measures fully account for sustained engagement failure and its variability, the buffering framework would be unnecessary. Conversely, superior predictive validity of time-dependent engagement metrics would support the present model.

Longitudinal studies comparing engagement-duration measures with conventional executive-function indices using mixed-effects modeling provide a direct empirical test of this prediction.

### 8.6 Empirical scope

These predictions are formulated at the level of behavioral dynamics and clinical phenomenology. Current neuroimaging and electrophysiological tools lack sufficient reliability and temporal resolution to serve as direct clinical indices of engagement buffering. This limitation reflects a measurement gap rather than a theoretical weakness.

Future work may integrate neurobiological measures—such as fMRI indices of network efficiency or EEG markers of attentional variability—as complementary correlates rather than primary validators (9, 39).

A critical falsification condition for the present framework would be evidence that disengagement occurs independently of cumulative load or demand rate, or that performance decline follows a strictly linear or gradual trajectory without evidence of threshold-dependent transition. Similarly, if re-engagement occurs symmetrically and without dependence on prior load accumulation, the assumption of metastable buffering dynamics would be weakened. These conditions provide clear criteria for distinguishing the present framework from models based solely on continuous modulation of arousal, effort, or resource availability.

### 8.7 Individual differences: ADHD subtypes and comorbid anxiety

The buffering framework generates specific predictions regarding individual variation in engagement dynamics across ADHD presentations and comorbid conditions that are empirically distinguishable from prior models.

Within ADHD, predominantly inattentive presentations are predicted to be characterized primarily by reduced operational capacity and accelerated load accumulation under low-salience conditions, producing early and frequent transitions to disengagement with relatively limited compensatory mobilization under urgency. Predominantly hyperactive-impulsive presentations are predicted to reflect narrow operational bandwidth with a strong preference for rapid state exit—consistent with GSF activation at low thresholds—producing impulsive transitions before cumulative load reaches high levels. Combined presentations are expected to show both features: reduced capacity, narrow bandwidth, and frequent transitions across both low- and high-salience conditions. These differential predictions can be tested by comparing time-to-disengagement curves, lapse clustering patterns, and urgency-driven recovery across DSM presentation specifiers, with the specific prediction that inattentive presentations will show steeper late-stage performance decline while hyperactive-impulsive presentations will show earlier and more frequent transitions independent of cumulative load.

Comorbid anxiety introduces a qualitatively distinct pattern of interaction. Anxiety is associated with heightened vigilance, elevated arousal, and increased monitoring of internal states (45), each of which may contribute to accelerated buffering load accumulation via heightened dBL/dt. The co-occurrence of ADHD and comorbid anxiety is therefore predicted to produce disproportionately shortened time-to-disengagement under emotionally loaded or evaluatively threatening conditions, relative to either condition alone. However, anxiety may also exert a partially compensatory stabilizing effect in some contexts, as heightened vigilance can increase the salience of task-relevant stimuli and temporarily reduce the switching tendency associated with reduced buffering reserve. This bidirectional prediction—anxiety amplifying load-driven collapse in most contexts while occasionally stabilizing engagement in threat-congruent or high-stakes settings—is empirically distinguishable from models that treat anxiety comorbidity as uniformly inhibitory or uniformly facilitative. Specifically, the OE/GSF framework predicts that ADHD+anxiety will show greater sensitivity to demand rate than ADHD-only groups, while showing attenuated performance differences in brief, evaluatively salient tasks that leverage anxiety-driven vigilance to partially stabilize engagement.

Furthermore, the framework predicts that individuals with higher baseline operational capacity—whether due to developmental maturation, pharmacological modulation, or supportive environmental scaffolding—will show longer time-to-disengagement, lower intra-individual variability, and more graceful (less abrupt) recovery following collapse. These parameters represent quantitative individual differences within the same constraint-level architecture, rather than qualitatively distinct regulatory mechanisms, suggesting that the framework can account for dimensional variation in ADHD severity without invoking additional constructs.

## 9 Conclusion

ADHD is best understood not as a failure of executive knowledge, motivation, or intelligence, but as a disorder of sustained engagement under cumulative demand. By conceptualizing engagement as a buffered mental state governed by Operational Economy—a regulatory constraint principle, not an energetic construct—and implemented through automatic, pre-volitional switching dynamics, the present framework reconciles core clinical phenomenology with time-dependent behavioral instability and partial treatment response.

This reframing preserves patient agency by distinguishing engagement collapse from voluntary withdrawal or motivational failure, while resolving longstanding paradoxes in ADHD research—such as intact initial performance alongside abrupt disengagement and pronounced context sensitivity. Importantly, the framework operates at the level of functional constraints, clarifying how engagement fails over time without presupposing specific neural mechanisms. The explicit differentiation from cognitive-energetic, state-regulation, neuroenergetic, and dynamical-systems accounts positions OE/GSF as a clinically tractable complement to these established frameworks, generating unique predictions regarding demand-rate sensitivity, asymmetric re-engagement, and pharmacological specificity.

By grounding ADHD in lawful buffering constraints and state transitions, the present account generates clear, falsifiable predictions and provides a principled foundation for future measurement and intervention strategies. Shifting the clinical focus from static capacity to engagement stability offers a coherent, humane, and clinically actionable path forward for understanding and treating ADHD.

## Data Availability

The original contributions presented in the study are included in the article/supplementary material. Further inquiries can be directed to the corresponding author.
